# Safe implementation of hand held steerable laparoscopic instruments: a survey among EAES surgeons

**DOI:** 10.1007/s13304-022-01258-w

**Published:** 2022-04-13

**Authors:** S. F. Hardon, A. M. Rahimi, R. R. Postema, E. Willuth, Y. Mintz, A. Arezzo, J. Dankelman, F. Nickel, T. Horeman, the EAES Technology Committee Study Group, L. Baldari, L. Boni, M. Chand, F. Ficuciello, H. Fuchs, T. Horeman, N. Inaki, R. Jimenez-Rodrigues, Y. W. Kim, L. Manfredi, S. Marconi, P. Mascagni, S. Perretta, M. Schijven, G. Mylonas, P. Myśliwiec, F. Nickel, W. Petz, C. Sagiv, J. A. Sánchez-Margallo, F. Sánchez-Margallo

**Affiliations:** 1 Department of Surgery, Amsterdam UMC–VU University Medical Center, Room ZH 7F005, De Boelelaan 1117, 1081HV Amsterdam, The Netherlands; 2 Department of BioMechanical Engineering, Delft University of Technology, Delft, The Netherlands; 3 Department of General, Visceral and Transplantation Surgery, Heidelberg University Hospital, Heidelberg, Germany; 4 Department of General Surgery, Hadassah-Hebrew University Medical Center, Jerusalem, Israel; 5 Department of Surgical Sciences, Università degli Studi di Torino, Turin, Italy; 6 Technology Committee, European Association of Endoscopic Surgery (EAES), Veldhoven, The Netherlands

**Keywords:** Laparoscopy, Robot-assisted surgery, Steerable instruments, Patient safety, Survey

## Abstract

The complexity of handheld steerable laparoscopic instruments (SLI) may impair the learning curve compared to conventional instruments when first utilized. This study aimed to provide the current state of interest in the use of SLI, the current use of these in daily practice and the type of training which is conducted before using SLI in the operating room (OR) on real patients. An online survey was distributed by European Association of Endoscopic Surgery (EAES) Executive Office to all active members, between January 4th and February 3rd, 2020. The survey consisted of 14 questions regarding the usage and training of steerable laparoscopic instruments. A total of 83 members responded, coming from 33 different countries. Twenty three percent of the respondents using SLI, were using the instruments routinely and of these 21% had not received any formal training in advance of using the instruments in real patients. Of all responding EAES members, 41% considered the instruments to potentially compromise patient safety due to their complexity, learning curve and the inexperience of the surgeons. The respondents reported the three most important aspects of a possible steerable laparoscopic instruments training curriculum to be: hands-on training, safe tissue handling and suturing practice. Finally, a major part of the respondents consider force/pressure feedback data to be of significant importance for implementation of training and assessment of safe laparoscopic and robotic surgery. Training and assessment of skills regarding safe implementation of steerable laparoscopic instruments is lacking. The respondents stressed the need for specific hands-on training during which feedback and assessment of skills should be guaranteed before operating on real patients.

## Introduction

Since the late 1980s, robot-assisted surgery (RAS) has been gaining ground and has become the standard of care for several complex surgical procedures [1–7]. One of the advantages of RAS is the use of (distally) articulating robotic instruments which increase the degrees of freedom. However, due to the high acquisition and operating costs of RAS, the desire for a more affordable alternative has increased [8–10]. These more affordable alternatives should benefit both the patient and the surgeon [11, 12]. Consequently, hand held steerable laparoscopic instruments (SLI) were developed, offering part of the benefits that the robotic instruments have, but with reduction of the overall costs [10, 13, 14]. In recent years, the usage of SLI increased predominantly as a result of the interest in single-incision laparoscopy and the improved ergonomics for the surgeon’s hand compared to conventional laparoscopic instruments [15–18].

The benefits and drawbacks of steerable instruments have been extensively reported in previous studies [15, 18–20]. Steerable instruments improve the range of motion and increase the degrees of freedom. Furthermore, by increasing the degree of freedom the physical strain and musculoskeletal pain of the surgeon’s upper extremities decreased. [15, 21, 22]. Training courses with box trainers and virtual reality trainers using conventional laparoscopic instruments improve surgical skills [23–26]. However, the complexity of the SLI may result in a more shallow learning curve and, therefore, may require additional training to pursue mastery of skills. The angular amplification of the instrument tip and the more complex handle controls due to the increased degrees of freedom could overwhelm the surgeon and cause an initial longer training process [21, 27].

The aim of this study consisted of surveying members of the European Association of Endoscopic Surgery (EAES) to analyze and provide the current state of interest in the usage of steerable instruments. Moreover, we determined the current perceptions and use of the steerable instruments in daily practice and the type of training which has been conducted prior to using the instruments during laparoscopic surgery.

## Methods

An online survey was conducted using a questionnaire designed and distributed by the European Association of Endoscopic Surgery (EAES) Executive Office to all active members of the EAES. The survey was conducted between January 4th and February 3rd, 2020. The survey consisted of 14 questions, presented in Table 1, regarding the usage and training of steerable laparoscopic instruments. Furthermore, the questions were designed to determine the opinion regarding objective assessment of the surgical performance with steerable instruments. GraphPad (Prism 9.0.0, San Diego, California USA) was used for frequency distribution and to generate the graphs. This anonymous survey was exempt from IRB approval.

**Table 1:** Survey questions

Q1. Are you interested in using steerable (handheld) instruments during your procedure? (*yes/no/other*)
Q2. How often did you try a new steerable laparoscopic instrument in the operating room (OR)? (*0–5 times, 5–10 times, 10–20 times,* > *20 times*)
Q3. What was the name/brand of the instrument? By which company was it manufactured?
Q4. Did you receive any specific training in advance of using a steerable instrument in the OR? (*yes/no*)
Q5. If so, what type of training did you receive before using the instrument in the OR? (*Demonstration by instructor, theoretical (eLearning, instruction form, booklet), hands-on box training, VR training, Cadaver training, proctoring in the OR, other than the above mentioned, I did not receive any specific training*)
Q6. Based on your expert opinion, do you see any potential risks in using steerable instruments in complex laparoscopic procedures? (*yes/no/other*)
Q7. Please provide three aspects of training we should mainly focus on, in case of training with new steerable instruments:
Q8. Do you think it is important to use force/pressure data as measures for safe laparoscopic surgery during assessment in and outside the OR? (Please specify: *yes/no*)
Q9. Do you think it would be good or necessary to have tissue force/pressure data integrated during robot-assisted surgery (e.g., da Vinci) for training and/or for clinical routine? (Please specify: *yes/no*)
Q10. Do you actually use steerable instruments routinely? If so, during what kind of procedures it is most beneficial to use these compared to conventional laparoscopic instruments? (Please specify: *yes/no*)
Q11. What is your profession?
Q12. What is your nationality?
Q13. What is your gender?
Q14. If a course or training for safe use of steerable instruments was organized by the EAES during the 2020 congress in Poland, would you be interested in attending it? (*yes/no/other)*

## Results

A total of 83 (out of 3582) active members, of whom 80 were practicing surgeons, responded. The respondents originated from 33 countries, with the highest proportion from the Netherlands (14%), Italy (11%) and the United Kingdom (6%). The respondents held a position either as a surgeon (94%), a fellow (1%), or a resident (5%).

**Fig. 1:**
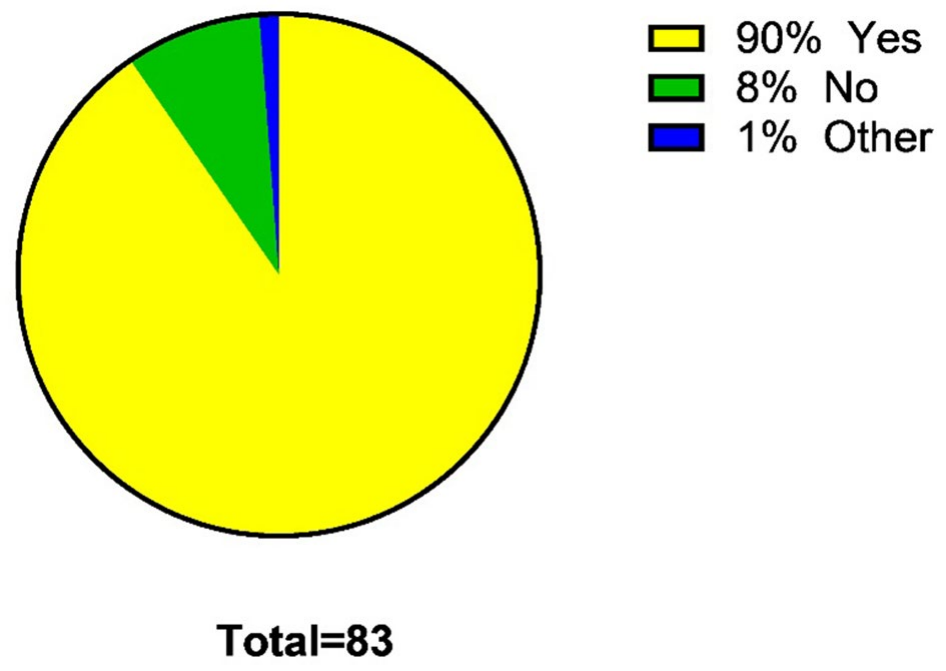
Percentage of respondents interested in steerable instruments

**Fig. 2:**
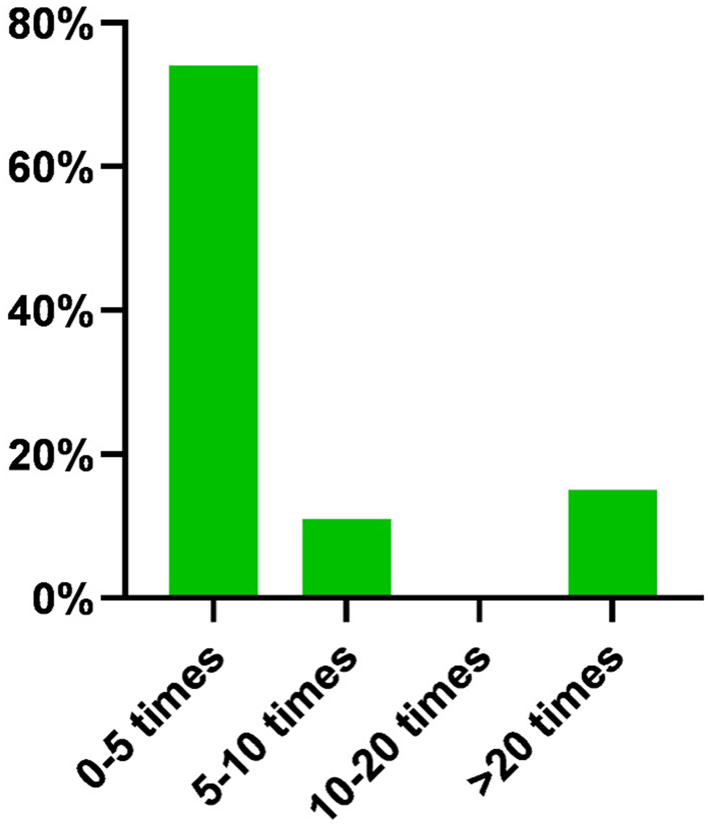
Amount of times the steerable laparoscopic instruments were used on patients by the respondents using SLI

The vast majority of 75 participants (90%) was interested in using SLI during surgery in the OR (Fig. 1). Of the respondents, 61 (74%) had previously utilized a new SLI in the OR, but less than 5 times, 9 (11%) responded 5–10 times, and 12 (15%) confirmed they had used SLI more than 20 times in the OR (Fig. 2).

**Fig. 3:**
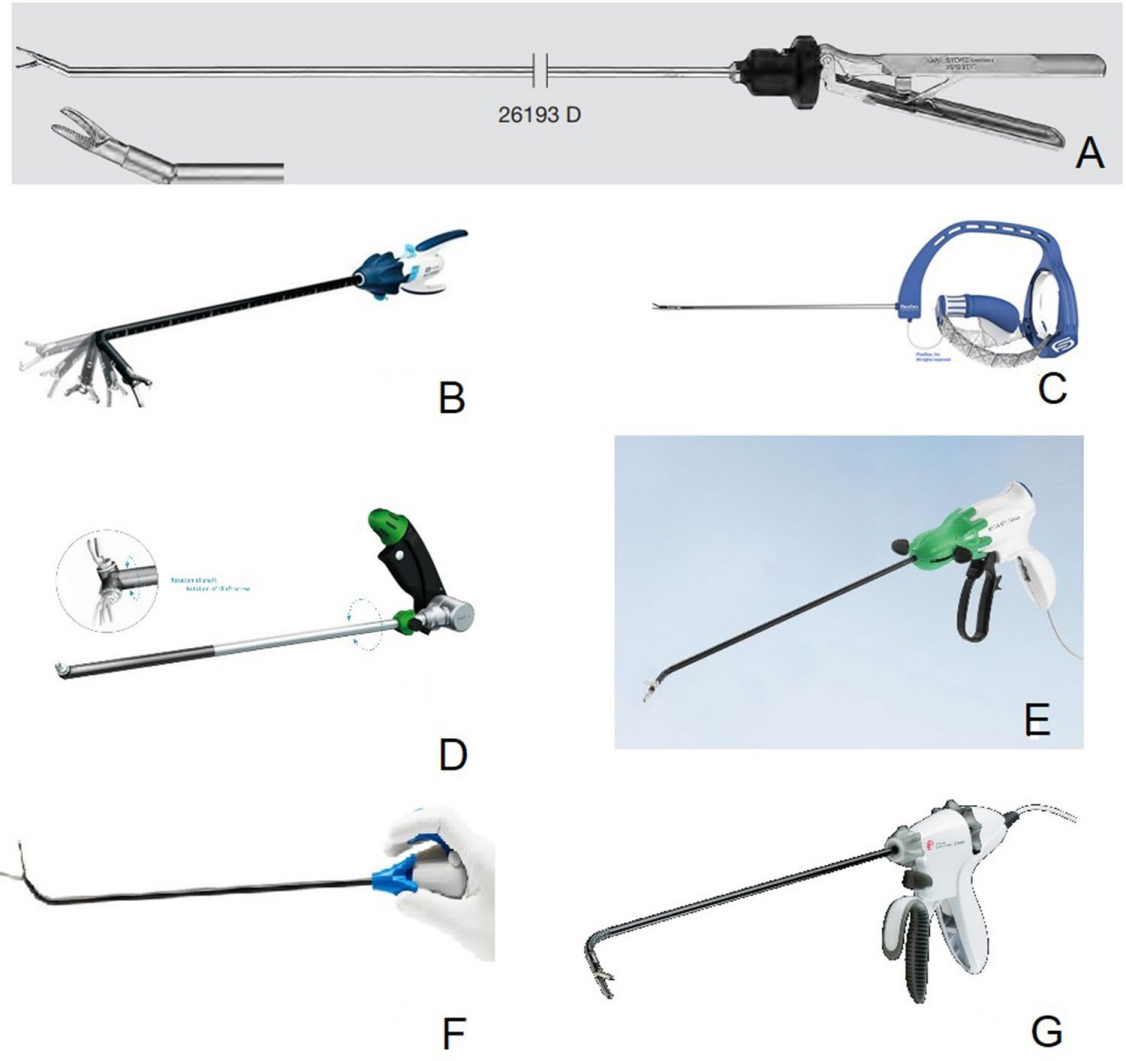
Most commonly reported SLI

Of all surgeons who used SLI in the OR, 23% used it routinely, and most commonly to perform colorectal surgery procedures. When asked about the brand and company of the previously used SLI, the members responded with 23 different brands and companies, of which 17% Karl Storz SE & Co. KG (Tuttlingen, Germany), 17% Medtronic plc (Dublin, Ireland), 14% Alphatron Surgical B.V. (Rotterdam, The Netherlands) and 10% Tuebingen Scientific Medical GmbH (Tuebingen, Germany) were the most common (Fig. 3).

**Fig. 4:**
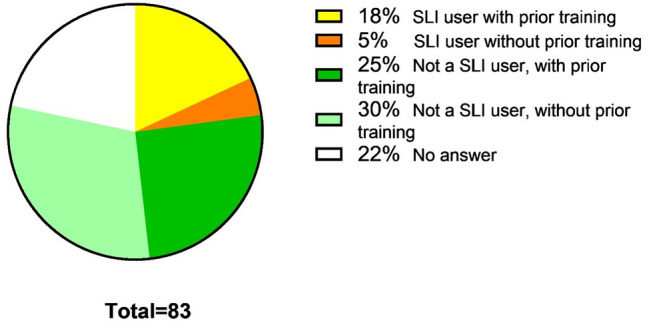
Percentage of members which had received prior training

**Fig. 5:**
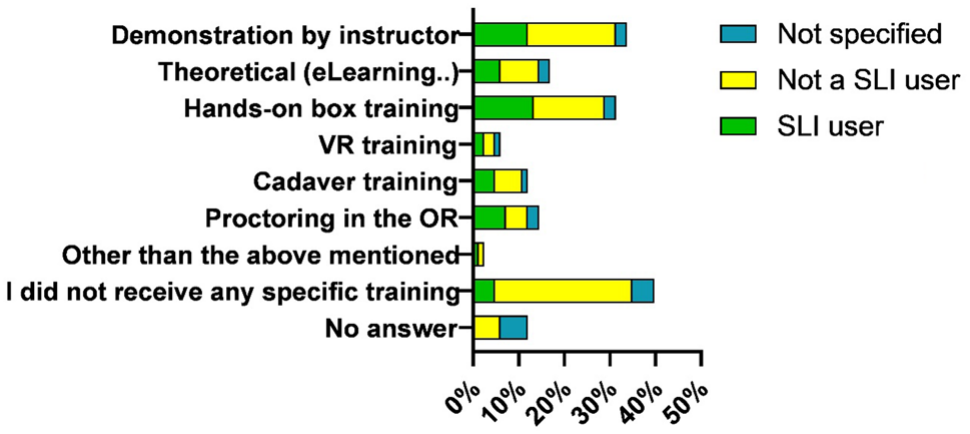
Types of received training before using the instrument without supervision in the OR on patients. Combination of options are possible

Twenty three percent of the respondents using SLI, were using SLI routinely and of this group twenty one percent had not received any specific training in advance of using the SLI in the OR (Fig. 4). If the participant had received training, the three most common types of training consisted of: demonstration by instructor (34%), hands-on box training (31%) and theoretical training (eLearning, instruction form or booklet) (17%) (Fig. 5). Regarding potential risks of SLI concerning patient safety, 46% of the respondents foresaw no risk. Yet, 41% of respondents considered patient safety to be compromised with the introduction of SLI. The most frequent argument given for no risk was that before using SLI training is essential and only a good trained surgeon should perform surgery with steerable instruments and thus reducing the risk. The most common mentioned reason for potential risks consisted of the difficult and shallow learning curve and the lack of experience of the surgeons.

The three most reported aspects of training for steerable laparoscopic instruments were tissue handling, hands-on training and suturing practice. Fifty respondents (60%) reported that force/pressure data, with regard to tissue handling skills, is of significant importance for the assessment of safe laparoscopic surgery in and outside of the OR. In addition, 56 out of 83 respondents (67%) answered that it is necessary to have force/pressure data integrated during robotic surgery for training and/or clinical routine.

Below listed the most frequently mentioned manufacturers and instruments (Fig. 3), a short example that was provided by the respondents and the number (%) of users.

1. Karl Storz SE & Co. KG (Tuttlingen, Germany) (Fig. 3A), Articulating Needle Holder (17%).

2. Medtronic plc (Dublin, Ireland) (Fig. 3B), Covidien SILS™ Stitch articulating suturing device (17%).

3. Alphatron Surgical B.V. (Rotterdam, The Netherlands) (Fig. 3C), FlexDex system (14%).

4. Tuebingen Scientific Medical GmbH (Tuebingen, Germany) (Fig. 3D), Radius surgical system (10%).

5. B. Braun (Melsungen, Germany) (Fig. 3E), Caiman® 5 Articulating Maryland (3%).

6. Deam Products B.V. (Roden, The Netherlands) (Fig. 3F), LaproFlex (3%).

7. Johnson & Johnson (New Brunswick, New Jersey, United States) (Fig. 3G), ENSEAL G2 Articulating Tissue Sealer (3%).

## Discussion

This study, conducted with an questionnaire among EAES members, provided insight and perceptions regarding the implementation and adoption of SLI. Moreover, the results indicate to what extent specific skills for SLI have been trained and assessed before operating on real patients. The vast majority of the respondents had interest in using steerable instruments. However, only a small fraction was using the steerable laparoscopic instruments routinely. Furthermore, twenty one percent of this group did not receive any form of prior training regarding steerable laparoscopic instruments. The respondents that did receive training, replied that the training consisted mainly of demonstration by an instructor or hands-on box training.

The majority of responding EAES members raised concerns on risks for patient safety, due to a shallow learning curve and the inexperience of the surgeon, when introducing the use of SLI. With this in mind the respondents determined the three most important aspects of steerable laparoscopic instruments to be: safe tissue handling, hands-on training and suturing practice. Furthermore, there was consensus among the respondents on the use of force-based feedback on tissue handling skills and its importance for the assessment of safe laparoscopic surgery. This same consensus, regarding the force/pressure data, consists for robotic surgery training.

Previous studies have compared conventional and steerable laparoscopic instruments in performance, ergonomics, learning curve and (the lack of) training [15, 18, 27, 28]. Santos et al. [28] compared two groups of medical students (*n* = 45) with one group receiving laparoscopic training with conventional instruments, and the other group receiving single-incision laparoscopy training with articulating instruments. The groups performed laparoscopic and single incision laparoscopic peg transfer and pattern cutting tasks. The learning curve for the articulating instruments was longer compared to conventional instruments and the performance with conventional instruments was superior. Besides, Uysal et al. (2020) [27] performed a randomized cross-over study with fifty laparoscopic novices comparing conventional and articulating laparoscopic instruments in completing the European training in basic laparoscopic urological skills (E-BLUS). The conventional laparoscopic instruments group had a better performance, with the longer learning curve of the articulating instruments being one of the possible reasons for this outcome. Corker et al. [18] compared articulating versus conventional instruments and also the combination of both instruments. This study consisted of three groups of surgeons with different combinations of instruments (*n* = 21): two articulating instruments, two conventional, and a combination of both. The groups performed a peg transfer task and a pattern-cutting task. The group with one articulating and one conventional laparoscopic instrument performed best in the peg transfer task. Finally, Sánchez-Margallo et al. [15] performed a study comparing six laparoscopic surgeons while using both conventional and a handheld motor-driven laparoscopic needle holder during three different suturing tasks. In this study, no significant differences were observed between the instruments regarding performance. However, the SLI did result in better outcomes with regards to ergonomics.

A limitation of this study is possible participation bias. EAES members who are more interested in SLI are more likely to participate in the survey, this also follows from the responses as ninety percent of the respondents was interested in SLI. Furthermore, a relatively small number of EAES members participated in the survey. A strength of this survey is the diversity in the country of origin of the respondents providing a variety in experience. Moreover, the respondents had experience with a large variety of SLI brands and, therefore, contributing to the survey with a more diverse experience.

Mainly driven by the large interest in SLI combined with an indicated uncertainty in the ability to safely control the instruments during complex surgical tasks as tissue manipulation or suturing, it is advisable to develop training initiatives for technical skills training and assessment specific for SLI. This training should allow residents and surgeons to practice with any type of SLI in a realistic setting using for example a physical hands-on trainer to overcome the learning curve for these instruments. Based on our previous results [26, 29, 30] and based on the outcomes of this present study, the training system ideally provides objective performance feedback that reflect safe tissue handling. Doing so, surgeons can train new specific technical skills to control SLI. Adequate assessment of tissue interaction forces and unintentional errors at the end of training may reduce exerted forces and minimize tissue trauma when first utilizing SLI in real patients. [26, 30, 31]

## Conclusion

Training and assessment of technical skills for safe use of steerable laparoscopic instruments is lacking. Despite the large interest in these instrument, there are limited training initiatives and few surgeons have been trained sufficiently. According to the responding EAES members in this study, hands-on box trainers must be used for training of specific technical skills. Moreover, the respondents stressed the importance of objective force-based feedback to train safe handling of tissues before operating on real patients.
